# Intravenous Dexmedetomidine Has Synergistic Effect on Subarachnoid Block with Hyperbaric Bupivacaine

**DOI:** 10.7759/cureus.6051

**Published:** 2019-11-01

**Authors:** Aamir Furqan, Muhammad Usman Mohsin, Muhammad Kaleem Sattar, Ali A Khan, Muhammad Shahid, Aatir Fayyaz

**Affiliations:** 1 Anaesthesia and Intensive Care, Chaudhry Pervaiz Elahi Institute of Cardiology, Multan, PAK; 2 Anaesthesia and Intensive Care, Nishtar Medical University, Multan, PAK; 3 Anaesthesia, DHQ Teaching Hospital, Sahiwal, PAK; 4 Anaesthesia, The Children's Hospital & the Institute of Child Health, Multan, PAK

**Keywords:** dexmedetomidine, anesthesia, bupivacaine, subarachnoid, intravenous, bolus, infusion

## Abstract

Objective

To assess the effect of intravenous dexmedetomidine on subarachnoid anesthesia with the help of hyperbaric bupivacaine when administered as a bolus or as an infusion.

Materials and methods

This randomized control trial was conducted at the Department of Anesthesia, Nishtar Hospital, Multan, Pakistan, from January 2017 to December 2018. Seventy patients were enrolled in the study. Patients were segregated into three groups. At the T10 level, a sensory blockade was noted. The motor blockade was also periodically measured until a modified Bromage score of three was achieved. The depth of sedation was measured with the help of the Ramsay Sedation Scale score. Oxygen saturation and other factors were also measured and recorded. Nausea, vomiting, diarrhea, and pruritus were the adverse effects noted during the study. To check and compare the statistical differences among the variables from different groups, the Chi-square test and analysis of variance test were performed. A probability (p) value of <.05 was considered statistically significant.

Results

The duration of the sensory blockade was shortest in the control group receiving only bupivacaine (Group B) and longest in the group receiving bupivacaine plus dexmedetomidine as a single bolus (Group BDexB; p: <.001). The time of complete sensory and motor recovery was longest in Group BDexB and shortest in Group B. The difference was statistically significant (p: <.001). The Ramsay score was >2 (i.e., 3 or 4) in five patients from Group B, 19 from Group BDexB, and 17 from the group receiving intrathecal bupivacaine plus dexmedetomidine as an infusion (Group BDexI). Between these groups, a statistically significant difference was found (p: <.001).

Conclusions

Intravenous administration of dexmedetomidine as either a bolus or infusion prolonged the duration of the sensory and motor blockade.

## Introduction

In lower limb and lower abdominal surgeries, subarachnoid anesthesia is a widely used method for providing regional anesthesia. To provide a prolonged motor and sensory blockade, multiple adjuvants have been utilized, including α-2 agonists, opioids, and 0.5% hyperbaric intrathecal bupivacaine [1]. Intrathecal, oral, or intravenous administration of an α-2 adrenoreceptor agonist like clonidine is associated with providing an extended duration of spinal anesthesia [2,3]. Dexmedetomidine, being free from the side effect of respiratory depression, can be used as an adjuvant in certain clinical settings [4,5]. Dexmedetomidine is an α-2-adrenoreceptor agonist and is a more selective drug. It has higher α-2 and α-1 activity as compared to clonidine [6]. Dexmedetomidine has analgesic, amnestic, and sedative properties [7]. Dexmedetomidine and clonidine have both been associated with prolongation of sensory as well as a motor blockade in some previous studies [8-10].

Multiple studies support the effectiveness of intravenous as well as intrathecal use of dexmedetomidine to prolong the sensory and motor blockade when administered after inducing spinal anesthesia. In this study, we evaluate the effect of intravenous dexmedetomidine at a dose of 1 µg/kg when administered as a single bolus or an infusion at a dose of 0.5 µg/kg. The efficacy was evaluated based on sensorimotor effects following administration after subarachnoid anesthesia with the help of hyperbaric bupivacaine (12.5 mg). Similarly, the adverse-effect profile was also evaluated between the two different regimens at the same dose of dexmedetomidine. Even though the efficacy of dexmedetomidine has been studied previously in multiple international studies, very little has been reported locally regarding the sensorimotor effect of different regimens of dexmedetomidine.

## Materials and methods

**Figure 1:**
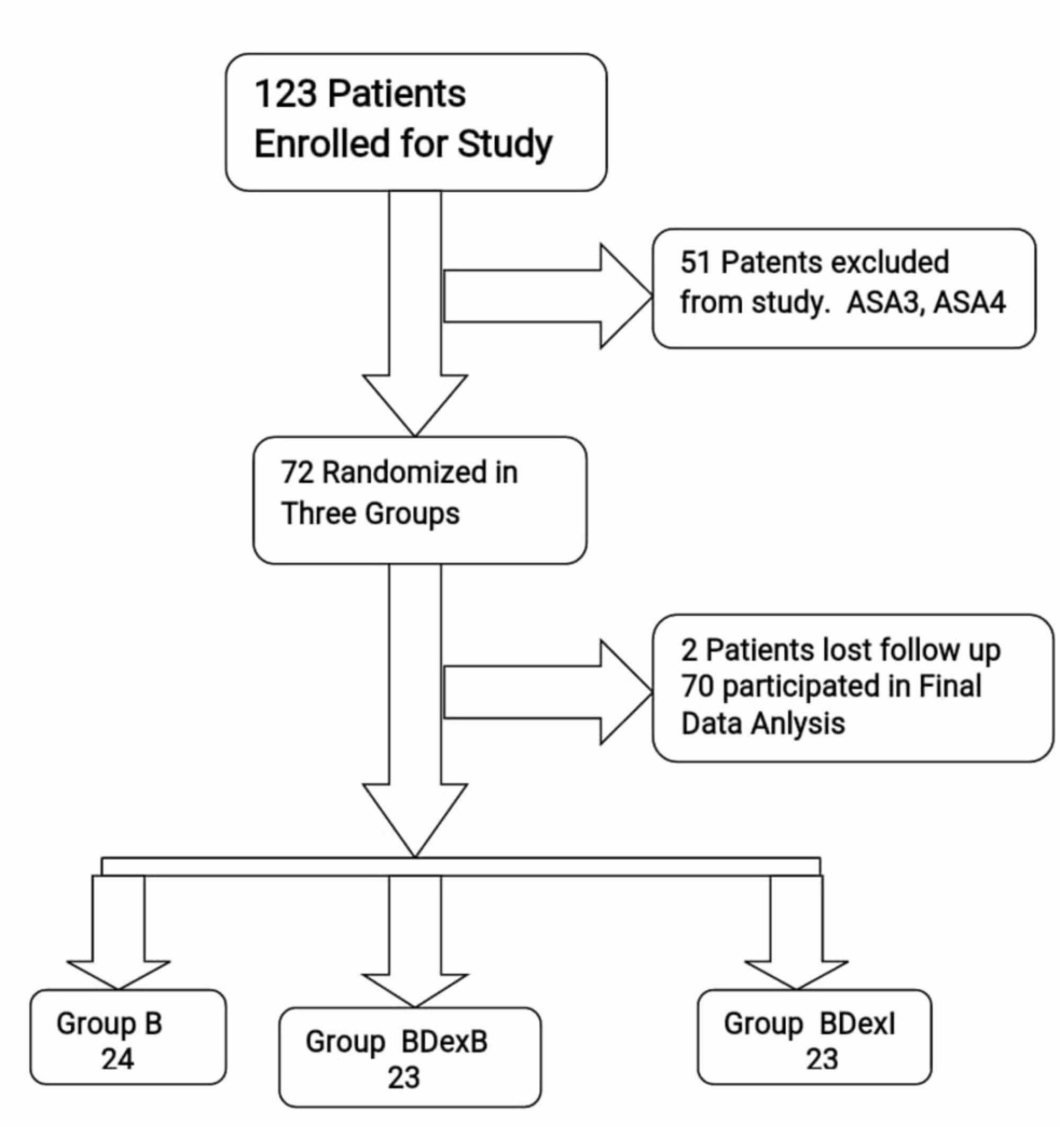
Flow sheet

This randomized control trial was performed at the Department of Anesthesia, Nishtar Hospital, Multan, Pakistan, from January 2017 to June 2018. Ethical approval was obtained from the hospital ethics committee. A total of 70 patients, aged 18 to 65 years, enrolled for elective lower limb surgery under subarachnoid anesthesia in the supine position. One or two patients belonging to the American Society of Anesthesiologists (ASA) Physical Status class (Figure 1) were also included. The sample size was calculated based on the reference study conducted by Kavya et al. [11]. All the procedures for preparing the mixtures of the drug being administered and administration of the drug to induce subarachnoid block and recording of sensorimotor effects of spinal anesthesia were evaluated by the researcher himself. 

A non-probability consecutive sampling technique was used to collect the sample. All the patients were randomly enrolled into one of the three groups. The control group received only 2 ml bupivacaine (Group B) in intrathecal space. In the bupivacaine plus dexmedetomidine (single bolus; BDexB) Group, the patients received 2 ml bupivacaine intrathecal and a bolus of 20 ml of normal saline mixed with dexmedetomidine 1 μg/kg in 10 minutes, followed by 20 ml of normal saline for the next 60 minutes. In the BDexI Group, the patients received intrathecal 2 ml bupivacaine plus 20 ml normal saline in 10 minutes, followed by dexmedetomidine infusion at a dose of 1 μg/kg.

After confirmation of cerebrospinal fluid free flow, intrathecal hyperbaric bupivacaine was administered via the L3 to L4 or L4 to L5 interspaces. The time of administration of intrathecal bupivacaine was set as the zero point. A sensory blockade was noted at the level of T10. The motor blockade was also periodically measured until a modified Bromage score of three was achieved. The Ramsay score was used to note the extent of sedation, and adverse effects such as hypotension, bradycardia, and nausea were also measured and recorded. Nausea, vomiting, diarrhea, and pruritus were the adverse effects noted during the study. The statistical analysis was done with the help of IBM SPSS Statistics for Windows, Version 23.0 (IBM, Armonk, NY). The frequency and percentages were calculated for all the qualitative variables, and the mean and standard deviation (SD) were calculated for the quantitative variables. To check and compare the statistical differences between the variables from different groups, Chi-square and ANOVA tests were performed. A probability (p) value of ≤.05 was deemed statistically significant.

## Results

All the groups were comparable in age, weight, gender distribution, and ASA status (p: >.05; Table 1). The time of onset of the sensory and motor blockades was not significantly different between the groups (p: >.05). The duration of the sensory blockade was longest in the BDexB Group and shortest in the B Group (p: <.001). The times for complete sensory and motor recovery were shortest in the B Group and longest in the BDexB Group; a statistically significant difference was found (p: <.001). The Ramsay score was >2 (i.e., 3 or 4) in five patients from Group B, in 19 patients from Group BDexB, and in 17 patients from the Group BDexI. A statistically significant difference was found (p: <.001) among all three groups. The rest of the patients in all groups had a Ramsay score of two (Table 2). The incidence of adverse effects such as hypotension, bradycardia, nausea, and peripheral capillary oxygen saturation (SpO2) of <95% was similar in all groups, and no statistically significant difference was observed (p: >.05; Table 3).

**Table 1:** Patient groups Data are entered as mean ± standard deviation (SD) unless otherwise mentioned. Group B: group receiving only bupivacaine; Group BDexB: group receiving bupivacaine plus dexmedetomidine as a single bolus; Group BDexI: group receiving intrathecal bupivacaine plus dexmedetomidine as an infusion ASA I/II: American Society of Anaesthesiologists physical status classification score; SD: standard deviation

Variable	Group B (n = 24)	Group BDexB (n = 23)	Group BDexI (n = 23)	P-value
Age, years	38.04 (±10.67)	35.39 (±10.79)	38.22 (±10.03)	.594
Weight, kg	53.75 (±11.69)	62.26 (±15.19)	55.95 (±13.31)	.088
Male/female	12/12	11/12	16/7	.261
ASA I/ASA II	15/9	17/6	15/8	.687

**Table 2:** Patient-group parameters Data are entered as mean ± standard deviation (SD) unless otherwise mentioned. Group B: group receiving only bupivacaine; Group BDexB: group receiving bupivacaine plus dexmedetomidine as a single bolus; Group BDexI: group receiving intrathecal bupivacaine plus dexmedetomidine as a bolus plus infusion Min: minutes; SD: standard deviation

Parameter	Group B (n = 24)	Group BDexB (n = 23)	Group BDexI (n = 23)	P-value
Onset of sensory blockade, min	2.3 (±0.2)	2.2 (±0.3)	2.3 (±0.2)	>.05
Duration of sensory blockade, min	131.08 (±14.52)	171.43 (±22.89)	163.43 (±16.54)	
Complete sensory recovery, min	213.02 (±28.45)	301.69 (±32.05)	286.09 (±19.43)	
Onset of motor blockade, min	2.1 (±0.4)	2.3 (±0.2)	2.2 (±0.3)	>.05
Motor recovery, min	227.71 (±19.02)	332.91 (±16.66)	312.22 (±22.68)	
Ramsey sedation score (2/3 or 4)	19/5	4/19	6/17	

**Table 3:** Adverse effects observed in patient groups Group B: group receiving only bupivacaine; Group BDexB: group receiving bupivacaine plus dexmedetomidine as a single bolus; Group BDexI: group receiving intrathecal bupivacaine plus dexmedetomidine as an infusion. Numbers in parentheses are the percentage figures SpO2: peripheral capillary oxygen saturation

Adverse effect	Group B (n = 24)	Group BDexB (n = 23)	Group BDexI (n = 23)	P-value
Hypotension	6 (25)	2 (8.7)	2 (8.7)	.181
Bradycardia	2 (8.3)	4 (17.4)	5 (21.7)	.435
Nausea	1 (4.2)	3 (13)	2 (8.7)	.554
SpO2 of <95%	1 (4.2)	4 (17.4)	4 (17.4)	.292

## Discussion

In this study, the effect of dexmedetomidine on subarachnoid anesthesia with intrathecal hyperbaric bupivacaine was evaluated when dexmedetomidine was given as a single bolus or infusion. In either method, the time required for the beginning of a sensory block was similar and comparable to results previously reported in other studies [9,12,13]. However, Harsoor et al. suggested different results and showed that dexmedetomidine was associated with a shorter time required for the onset of the sensory block as compared to the control group [5]. In this study, it is evident that the mean time taken by the two-segment regression or recovery of sensory block was also prolonged. This is in accordance with some previous studies [5,14-16]. The beginning of the motor blockade was comparable in all three groups in this study. Another study reported results similar to our study in terms of a comparable time for motor-block onset [8], while dexmedetomidine was associated with shortening the time necessary for the motor-block onset by a duration of one minute [17].

In both groups receiving dexmedetomidine, sensory recovery was comparable to the control group. This aligns with the conclusion made by previous studies [8,10]. For either the bolus or bolus plus infusion, the motor blockade induced by dexmedetomidine was comparable. Similar results have been shown in some past studies [5,10,14,17,18]. In other previous studies, the time taken to return to a modified Bromage scale of one was taken as the duration of the motor blockade [12,15,19,20]. This study showed that dexmedetomidine did not alter the time required by the motor blockade to return to a modified Bromage scale of zero. Some previous studies have deduced similar results as dexmedetomidine did not prolong the duration of the motor blockade in these studies. The prolongation of the motor blockade seen in this study can be attributed to the fact that we took a modified Bromage scale of zero as the endpoint. In this study, there were three groups: Group B received intravenous normal saline; Group BDexB was administered intravenous dexmedetomidine as a single bolus; Group BDexI was administered dexmedetomidine as an infusion. The post-hoc analysis found no statistically significant difference in the two groups receiving dexmedetomidine.

Only one similar study has been reported previously regarding the effect of dexmedetomidine as a single bolus or as a bolus plus infusion. As in our study, a similar study found no difference between the two groups administered with dexmedetomidine [11]. Further studies are required to evaluate the difference between single bolus and bolus plus infusion regimens of dexmedetomidine. As far as the sedation was concerned, it was monitored with the help of a six-point Ramsay score. The sedation scores in this study were higher in patients receiving dexmedetomidine, but the reversal of sedation was easy. These findings were also present in other studies where dexmedetomidine was used [7]. Similarly, oxygen saturation also did not drop significantly in this study and was easily treated with the help of oxygen supplementation. Similar results were found in a previous study where no patient developed desaturation of oxygen with the use of dexmedetomidine [21,22].

Our study was not without limitations. We included only those patients who needed lower limb surgery with ASA I and II in our study. More trials are needed on other types of surgeries and additional ASA grades. These studies would provide more clarity regarding the efficacy of dexmedetomidine on intrathecal bupivacaine.

## Conclusions

Our study concludes that the intravenous administration of dexmedetomidine as either a bolus or an infusion prolongs the duration of the sensory and motor blockade.

## Appendices

Response to Reviewers observations

1.      All corrections are made according to reviewers comments

2.     The flow sheet is added

3.     The rationale of the study is rephrased

## References

[1] Pitkänen M (2009). Spinal (subarachnoid) blockade. Cousins and Bridenbaugh’s neural blockade in clinical anesthesia and pain medicine.

[2] Rhee K, Kang K, Kim J, Jeon Y (2003). Intravenous clonidine prolongs bupivacaine spinal anesthesia. Acta Anaesthesiol Scand.

[3] Bonnet F, Buisson VB, Francois Y, Catoire P, Saada M (1990). Effects of oral and subarachnoid clonidine on spinal anesthesia with bupivacaine. Reg Anesth.

[4] Reves JG, Glass PS, Lubarsky DA, McEvoy MD, Martinez-Ruiz R (2010). Intravenous anesthetics. Miller’s Anesthesia Volumes 1 and 2, 7th Edition.

[5] Harsoor S, Rani DD, Yalamuru B, Sudheesh K, Nethra S (2013). Effect of supplementation of low dose intravenous dexmedetomidine on characteristics of spinal anaesthesia with hyperbaric bupivacaine. Indian J Anaesth.

[6] Kamibayashi T, Maze M (2000). Clinical uses of α2-adrenergic agonists. Anesthesiology.

[7] Hall JE, Uhrich TD, Barney JA, Arain SR, Ebert TJ (2000). Sedative, amnestic, and analgesic properties of small-dose dexmedetomidine infusions. Anesth Analg.

[8] Whizar-Lugo V, Gómez-Ramírez IA, Cisneros-Corral R, Martínez-Gallegos N (2007). Intravenous dexmedetomidine vs. intravenous clonidine to prolong bupivacaine spinal anesthesia. A double blind study. Anest en Mex.

[9] Tekin M, Kati I, Tomak Y, Kisli E (2007). Effect of dexmedetomidine IV on the duration of spinal anesthesia with prilocaine: a double-blind, prospective study in adult surgical patients. Curr Ther Res Clin Exp.

[10] Al-Mustafa MM, Badran IZ, Abu-Ali HM, Al-Barazangi BA, Massad IM, Al-Ghanem SM (2009). Intravenous dexmedetomidine prolongs bupivacaine spinal analgesia. Middle East J Anaesthesiol.

[11] Kavya UR, Laxmi S, Ramkumar V (2018). Effect of intravenous dexmedetomidine administered as bolus or as bolus-plus-infusion on subarachnoid anesthesia with hyperbaric bupivacaine. J Anaesthesiol Clin Pharmacol.

[12] Kaya FN, Yavascaoglu B, Turker G, Yildirim A, Gurbet A, Mogol EB, Ozcan B (2010). Intravenous dexmedetomidine, but not midazolam, prolongs bupivacaine spinal anesthesia. Can J Anaesth.

[13] Annamalai A, Singh S, Singh A, Mahrous DE (2013). Can intravenous dexmedetomidine prolong bupivacaine intrathecal spinal anesthesia?. J Anesth Clin Res.

[14] Hong JY, Kim WO, Yoon Y, Choi Y, Kim SH, Kil HK (2012). Effects of intravenous dexmedetomidine on low-dose bupivacaine spinal anaesthesia in elderly patients. Acta Anaesthesiol Scand.

[15] Lee MH, Ko JH, Kim EM, Cheung MH, Choi YR, Choi EM (2014). The effects of intravenous dexmedetomidine on spinal anesthesia: comparison of different dose of dexmedetomidine. Korean J Anesthesiol.

[16] Gupta K, Tiwari V, Gupta PK, Pandey MN, Agarwal S, Arora A (2014). Prolongation of subarachnoid block by intravenous dexmedetomidine for sub umbilical surgical procedures: a prospective control study. Anesth Essays Res.

[17] Elcıcek K, Tekın M, Katı I (2010). The effects of intravenous dexmedetomidine on spinal hyperbaric ropivacaine anesthesia. J Anesth.

[18] Dinesh CN, Sai Tej NA, Yatish B, Pujari VS, Mohan Kumar RM, Mohan CVR (2014). Effects of intravenous dexmedetomidine on hyperbaric bupivacaine spinal anesthesia: a randomized study. Saudi J Anaesth.

[19] Reddy VS, Shaik NA, Donthu B, Reddy Sannala VK, Jangam V (2013). Intravenous dexmedetomidine versus clonidine for prolongation of bupivacaine spinal anesthesia and analgesia: a randomized double-blind study. J Anaesthesiol Clin Pharmacol.

[20] Jung SH, Lee SK, Lim KJ (2013). The effects of single-dose intravenous dexmedetomidine on hyperbaric bupivacaine spinal anesthesia. J Anesth.

[21] Abdallah FW, Abrishami A, Brull R (2013). The facilitatory effects of intravenous dexmedetomidine on the duration of spinal anesthesia: a systematic review and meta-analysis. Anesth Analg.

[22] Niu XY, Ding XB, Guo T, Chen MH, Fu SK, Li Q (2013). Effects of intravenous and intrathecal dexmedetomidine in spinal anesthesia: a meta-analysis. CNS Neurosci Ther.

